# Supplementary figures and images for: QTL mapping for pod quality and yield traits in snap bean (Phaseolus vulgaris L.)
Source: Front Plant Sci. 2024 Aug 12;15:1422957. doi: 10.3389/fpls.2024.1422957 (PMC11345156; doi:10.3389/fpls.2024.1422957)

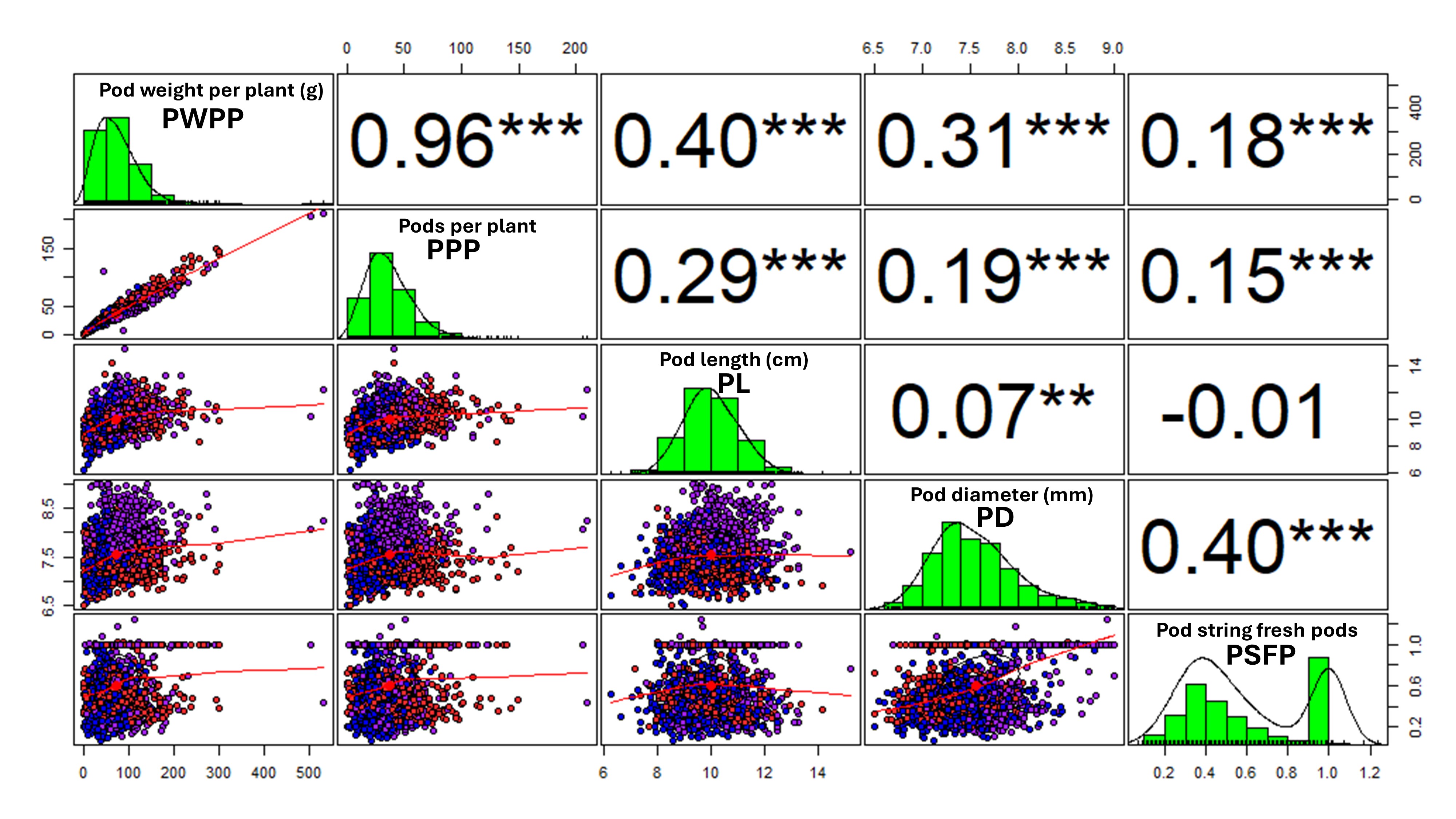

Supplement: Supplementary Figure 1 — Phenotypic correlations of quantitative traits between field data for all RILs at all field sites. Upper panels indicate Spearman correlation coefficients (r), while diagonal and lower panels represent distributions of the data among RILs. Point color represents location: Don Bosco (purple), Mariira (red), Kutus (blue). [file Image_1.jpeg]

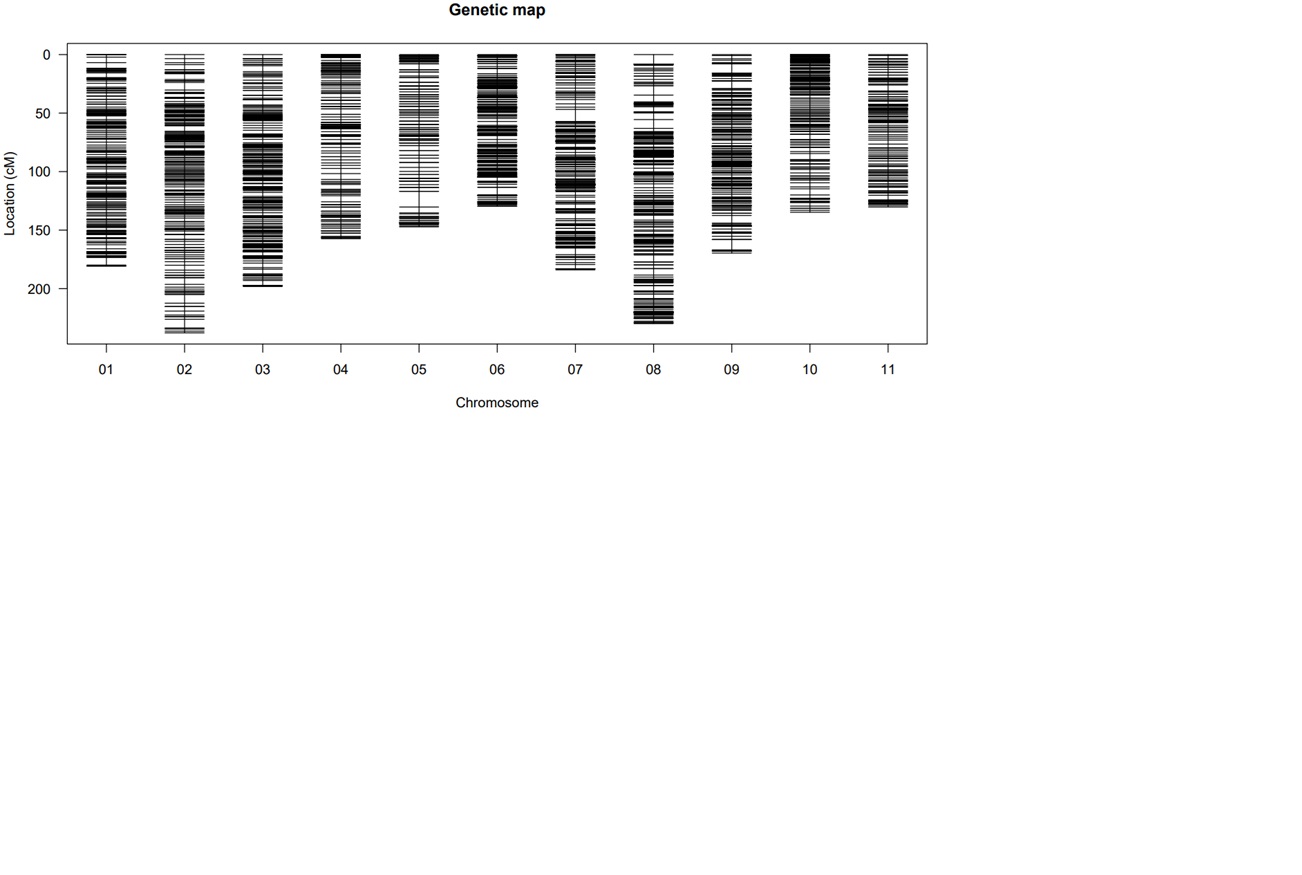

Supplement: Supplementary Figure 2 — Vanilla x MCM 5001 common bean genetic map derived from linkage analysis of 5,951 SNPs. [file Image_2.jpeg]
